# AKI-Pro score for predicting progression to severe acute kidney injury or death in patients with early acute kidney injury after cardiac surgery

**DOI:** 10.1186/s12967-024-05279-4

**Published:** 2024-06-16

**Authors:** Ying Su, Peng Wang, Yan Hu, Wen-jun Liu, Yi-jie Zhang, Jia-qi Chen, Yi-zhi Deng, Shuang Lin, Yue Qiu, Jia-kun Li, Chen Chen, Guo-wei Tu, Zhe Luo

**Affiliations:** 1 Cardiac Intensive Care Center, Zhongshan Hospital, Fudan University, No. 180 Fenglin Road, Xuhui District, Shanghai, 200032 China; 2 Department of Anesthesiology, Zhongshan Hospital, Fudan University, No. 180 Fenglin Road, Xuhui District, Shanghai, 200032 China; 3 Shanghai Key Laboratory of Lung Inflammation and Injury, Shanghai, China; 4 Department of Critical Care Medicine, Shanghai Xuhui Central Hospital, Zhongshan-Xuhui Hospital, Fudan University, Shanghai, China

**Keywords:** Acute kidney injury, AKI progression, Modified Furosemide responsiveness index, Furosemide stress test, Renal replacement therapy, Predictive score, Cardiac surgery

## Abstract

**Background:**

No reliable clinical tools exist to predict acute kidney injury (AKI) progression. We aim to explore a scoring system for predicting the composite outcome of progression to severe AKI or death within seven days among early AKI patients after cardiac surgery.

**Methods:**

In this study, we used two independent cohorts, and patients who experienced mild/moderate AKI within 48 h after cardiac surgery were enrolled. Eventually, 3188 patients from the MIMIC-IV database were used as the derivation cohort, while 499 patients from the Zhongshan cohort were used as external validation. The primary outcome was defined by the composite outcome of progression to severe AKI or death within seven days after enrollment. The variables identified by LASSO regression analysis were entered into logistic regression models and were used to construct the risk score.

**Results:**

The composite outcome accounted for 3.7% (n = 119) and 7.6% (n = 38) of the derivation and validation cohorts, respectively. Six predictors were assembled into a risk score (AKI-Pro score), including female, baseline eGFR, aortic surgery, modified furosemide responsiveness index (mFRI), SOFA, and AKI stage. And we stratified the risk score into four groups: low, moderate, high, and very high risk. The risk score displayed satisfied predictive discrimination and calibration in the derivation and validation cohort. The AKI-Pro score discriminated the composite outcome better than CRATE score, Cleveland score, AKICS score, Simplified renal index, and SRI risk score (all *P* < 0.05).

**Conclusions:**

The AKI-Pro score is a new clinical tool that could assist clinicians to identify early AKI patients at high risk for AKI progression or death.

**Graphical Abstract:**

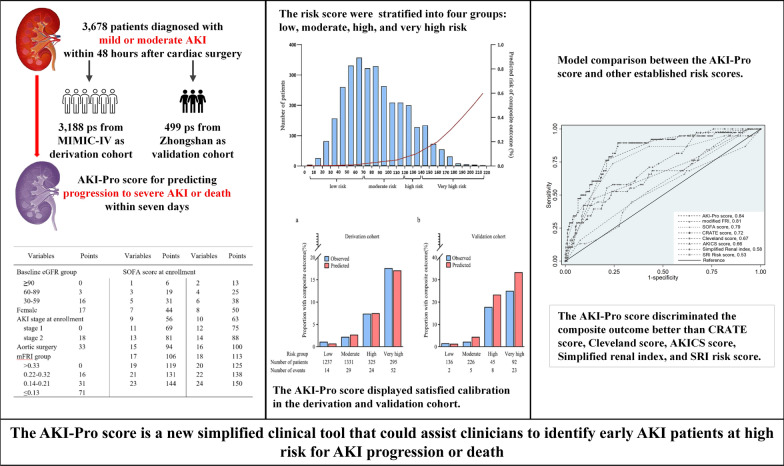

**Supplementary Information:**

The online version contains supplementary material available at 10.1186/s12967-024-05279-4.

## Background

Acute kidney injury (AKI) is one of the most common complications after cardiac surgery [1]. Although most cases of AKI following cardiac surgery are characterized by a mild and transient course, it is noteworthy that approximately 10–25% of patients initially presenting with mild AKI will experience a more severe stage or even dialysis during their postoperative hospitalization [2–4]. Once progression to severe AKI or requiring renal replacement therapy (RRT), there is a substantial increase in the long-term risks of developing chronic kidney disease (CKD), perioperative mortality by a magnitude of 3–8 times, prolonged hospital length of stay, and escalated healthcare costs [5–7]. Early identification of AKI progression could provide guidance for individualized clinical care, allocation of resources, and enrollment in AKI clinical trials.

Several investigators have dedicated their research efforts to exploring a range of novel biomarkers for predicting AKI progression. Biomarkers such as neutrophil gelatinase-associated lipocalin (NGAL), L-type fatty acid binding protein (L-FABP), interleukin (IL)-18, cystatin C (CysC), and tissue inhibitor of metalloproteinases (TIMP-2)/insulin-like growth factor-binding protein 7 (IGFBP7) have been identified [3, 8–10]. However, despite extensive investigation, the clinical utility of these biomarkers remains uncertain, and their availability for routine clinical use among physicians is limited, particularly in less developed countries. We recently proposed a new index, the modified furosemide responsiveness index (mFRI), as a surrogate for the furosemide stress test (FST). The mFRI incorporated a nonstandardized furosemide dose and demonstrated a significant association with the progression of AKI in two distinct cohorts [11].

Despite several studies exploring biomarkers or risk factors for AKI progression, there is a lack of clinical tools incorporating routine clinical perioperative variables available at the time of early AKI diagnosis to effectively identify high-risk patients who will progress to severe AKI, including dialysis or even death during hospitalization. In this study, we aimed to develop a new risk score to accurately predict the composite outcome in patients with initial mild and moderate AKI after cardiac surgery.

## Methods

The derivation cohort for this study was the retrospective Medical Information Mart for Intensive Care IV (MIMIC-IV) cohort, which consisted of data from diverse critical care units at Beth Israel Deaconess Medical Center between 2008 and 2019. Approval was obtained to access this database, as previously outlined [11]. The Zhongshan cohort, utilized for external validation, was an observational database that prospectively enrolled consecutive adult patients undergoing cardiac surgery in the cardiac surgical intensive care unit (CSICU) of Zhongshan Hospital, Fudan University, from May 2021 to November 2021. Ethical approval for this study was obtained from the Ethics Committee of Zhongshan Hospital, Fudan University (Shanghai, China; Approval No. B2021-390R), and the study was registered on ClinicalTrials.gov (ID: NCT04962412), as previously described [11].

### Patient cohort and data collection

Adult patients aged ≥ 18 years who experienced mild to moderate AKI (stage 1 or 2) within 48 h of cardiac surgery were included in both two cohorts. Patients were excluded if they met the following states: without AKI diagnosis or severe AKI (Kidney Disease Improving Global Outcomes (KDIGO) stage 3) diagnosis within 48 h after surgery, pre-existing history of CKD or RRT, kidney transplantation or other kidney diseases, re-operation or multiple operations during the hospitalization, not receive furosemide or receive furosemide after 24 h from AKI diagnosis, continuous furosemide infusion before or within 2 h after the first bolus dose, previously bolus furosemide used within 6 h of the first dose or repeated use of furosemide within 2 h after the first dose, pregnancy, missing baseline creatinine data, missing record of weight or urine output after furosemide administration, moribund state (likely to die within 24 h) (Fig. S1).

### Study variables and definitions

Demographic characteristics include age, gender, and body mass index (BMI). Comorbidities include diabetes mellitus (DM), hypertension, ischemic heart disease (IHD), and chronic obstructive pulmonary disease (COPD). Preoperative variables include baseline eGFR, nephrotoxic agent exposure, and diuretic exposure. Type of surgery includes coronary artery bypass grafting (CABG) only, Valve only, CABG and Valve, Aortic surgery, and other cardiac surgery. Clinical characteristics at enrollment include the AKI stage (KDIGO criteria stage 1 and stage 2), anemia, SOFA, mFRI.

AKI was defined based on the KDIGO criteria, which include serum creatinine and urine output criteria. The lowest serum creatinine (Scr) value available within three months before cardiac surgery was used as the baseline Scr. We estimated preoperative GFR using the modification of diet in renal disease (MDRD) equation. SOFA was derived from the monitoring data and laboratory parameters in ICU admission after surgery. The mFRI was calculated as the total urine output in 2 h divided by the intravenous bolus furosemide dose and body weight [mL/(mg·kg)/2 h] after AKI enrollment.

### Outcome measures

The primary outcomes were defined by the composite outcome of progression to severe AKI (from stage 1 to stage 3 or stage 2 to stage 3) or death within seven days after AKI enrollment.

### Model building and statistical analysis

Data were described as median (interquartile range) or mean (SD) for continuous variables and as frequencies and proportions for categorical variables. The student t test or Mann–Whitney U tests were used for continuous variables, whereas the χ^2^ test or Fisher’s exact tests were used for categorical variables, as appropriate. For missing data, multiple imputations with chain equations and an iteration of 10 times were used to estimate the missing data and were merged according to Rubin’s rules. And only the SOFA score has missing data (0.78%) in derivation cohorts.

Least Absolute Shrinkage and Selection Operator (LASSO) regression, which could avoid the multicollinearity and overfitting of variables, was applied to determine the significant features. This algorithm uniquely penalizes the absolute value of a regression coefficient by adding an L1 norm as a penalty, and selection regression for multivariable analyses, augmented with tenfold cross validation for internal validation [12]. The greater the penalization, the greater shrinkage of coefficients, and some coefficients can be shrunk to zero. The most predictive variables were selected by the lambda 1se rules and were entered into logistic regression models.

A risk score, called AKI-Pro score, was derived for each patient by summing the weighted score assigned to each variable based on its β coefficient in the above model. The AKI-Pro score was further categorized into four groups based on the distribution of the risk score: low risk, moderate risk, high risk, and very high risk. Differences between predicted and observed composite outcome rates were explored for different risk groups in derivation and validation cohorts. For model performance, we calculated the area under the receiver operating characteristic curve (AUC) and evaluated the calibration using the Hosmer–Lemeshow goodness of fit test. Furthermore, decision curve analysis (DCA) was used to evaluate the clinical utility value in this study. The concept of population net benefit (NB), which is based on threshold probability (Pt), is fundamental to decision curves [13].

Statistical analysis was performed using R version 4.2.1 (R Foundation for Statistical Computing) and STATA 17.0 for Windows (StataCorp Texas, USA). The "glmnet" R package was applied to perform the Lasso feature selection. All statistical tests were two-sided, and P < 0.05 was considered statistically significant.

## Results

### Clinical characteristics and univariate analysis

3188 patients from the MIMIC-IV database were included in the analysis as the derivation cohort, and 499 patients from the Zhongshan cohort were included in the analysis as the external validation cohort (Fig. S1). The baseline clinical, laboratory, and procedural variables of two cohorts were listed in Table 1. We used two independent cohorts with different baseline characteristics representing a heterogeneous AKI population. Statistically significant differences were observed between two cohorts in terms of age, gender, BMI, comorbidities, baseline renal function, type of surgery, and clinical characteristics at enrollment (all *P* < 0.05). In the derivation cohort, 56.1% was BMI ≥ 30 with a high incidence of hypertension (80.4%) and IHD (77.2%), while in the validation cohort, 7.6% was BMI ≥ 30 and 56.5% was BMI < 24.9. CABG (51.3%) was the primary type of surgery in the MIMIC-IV cohort, and Valve (57.7%) was the most in the Zhongshan cohort. Aortic surgery accounted for 5.7% and 18.6% in two cohorts, respectively.

**Table 1:** Baseline characteristics and study outcomes of derivation and validation cohort

	Derivation cohort (n = 3188)	Validation cohort (n = 499)	P value
Age, years	70[62,77]	63[54,70]	< 0.01
Gender, n (%)			< 0.01
Female	973 (30.5)	112 (22.4)	
Male	2215 (69.5)	387 (77.6)	
BMI_category, kg/m^2^, n (%)			< 0.01
< 24.9	391 (12.3)	282 (56.5)	
25.0–29.9	1008 (31.6)	179 (35.9)	
≥ 30	1789 (56.1)	38 (7.6)	
Comorbidities			
Diabetes mellitus, n (%)			< 0.01
No	2464 (77.3)	425 (85.2)	
Yes	724 (22.7)	74 (14.8)	
Hypertension, n (%)			< 0.01
No	625 (19.6)	225 (45.1)	
Yes	2563 (80.4)	274 (54.9)	
Ischemic heart disease, n (%)			< 0.01
No	726 (22.8)	440 (88.2)	
Yes	2462 (77.2)	59 (11.8)	
COPD, n (%)			< 0.01
No	2885 (90.5)	497 (99.6)	
Yes	303 (9.5)	2 (0.4)	
Preoperative			
Baseline creatinine,μmol/L	79.6(70.7,97.2)	88(75,102)	< 0.01
Baseline eGFR, ml/min/1.73m2	79.2(63.9,98.8)	77.1(63.7,91.3)	< 0.01
Baseline eGFR group, n (%)			0.049
> 90	1040 (32.6)	131 (26.3)	
60–90	1518 (47.6)	270 (54.1)	
30–60	630 (19.8)	98 (19.6)	
Exposure to at least one nephrotoxic agent^a^, n (%)	726 (22.7)	173 (34.7)	< 0.01
Antibiotics	168 (5.2)	28 (5.6)	
Antivirals	5 (0.15)	0(0)	
NSAIDS	139 (4.3)	61 (12.2)	
ARBs/ACE inhibitor	481 (15)	121 (24.2)	
Other drugs	7 (0.2)	1 (0.2)	
Preoperative diuretic exposure, n (%)			< 0.01
No	2608 (81.8)	183 (36.7)	
Yes	580 (18.2)	316 (63.3)	
Type of surgery, n (%)			< 0.01
CABG only	1636 (51.3)	56 (11.2)	
Valve only	752 (23.6)	288 (57.7)	
CABG and valve	553 (17.3)	34 (6.8)	
Aortic surgery	183 (5.7)	93 (18.6)	
Other cardiac surgery	64 (2.0)	28 (5.6)	
Clinical characteristics at enrollment			
AKI stage at enrollment, n (%)			< 0.01
Stage 1	1291 (40.5)	442 (88.6)	
Stage 2	1897 (59.5)	57 (11.4)	
Hemoglobin below 9 g/dL, n (%)			< 0.01
No	2163 (67.8)	370 (74.1)	
Yes	1025 (32.2)	129 (25.9)	
SOFA score	5(4,7)	8(7,10)	< 0.01
Vasopressor use, n (%)			< 0.01
No	2748 (86.2)	208 (41.7)	
Yes	440 (13.8)	291 (58.3)	
Furosemide dose, mg/kg	0.22(0.19,0.26)	0.31(0.25,0.4)	< 0.01
2 h urine output, mL	375(235,550)	275(170,450)	< 0.01
mFRI, mL/(mg·kg)/2 h	0.21(0.13,0.32)	0.2(0.12,0.33)	0.34
Outcomes			
Composite outcome, n (%)	119(3.7)	38(7.6)	
Progression to severe AKI, n (%)	112(3.5)	35(7)	
AKI requiring dialysis, n (%)	22(0.7)	17(3.4)	
ICU/hospital death, n (%)	14(0.4)	7(1.4)	
Death did not reach severe AKI, n (%)	7(0.2)	3(0.6)	

AKI, acute kidney injury; ICU, intensive care units; COPD, Chronic Obstructive Pulmonary Disease; GFR,  Glomerular Filtration Rate; CABG, Coronary Artery Bypass Grafting; NSAIDS, non-steroidal anti-inflammatory drugs; ACEI/ARB,  angiotensin-converting enzymr inhibitor/angiotensin receptor blocker; SOFA, Sequential Organ Failure Assessment; mFRI, modified Furosemide Responsiveness Index

^a^Some patients were exposed to more than one nephrotoxic agent. Antibiotics: aminoglycosides, vancomycin; Antivirals: acyclovir, ganciclovir; Other drugs: cisplatin, mannitol

Categorical variables were presented as frequency rates and percentages, continuous variables were expressed as median (IQR)

The incidence of composite outcome within seven days after enrollment occurred in 119(3.7%) of patients in the derivation cohort (112 patients developed severe AKI, 22 of whom received RRT, 7 of whom died, an additional 7 deaths occurred in patients who did not reach severe AKI), and the incidence composite outcome occurred in 38(7.6%) of patients in the validation cohort (35 patients developed severe AKI, 17 of whom received RRT, 4 of whom died, an additional 3 deaths occurred in patients who did not reach severe AKI) (Table 1). Univariate analysis for clinical and laboratory characteristics comparison between the composite and non-composite outcome at the time of enrollment is shown in Table S1. Many parameters were found to be significantly different between the composite outcome and non-composite outcome, such as baseline eGFR, type of surgery, mFRI, SOFA, and AKI stage (all *P* ≤ 0.05).

### Feature determination and risk score construction

Nineteen variables were included in the LASSO regression. After LASSO regression selection (Fig. S2), six independently significant predictors of the composite outcome were included in the risk model. These predictors included female, baseline eGFR, aortic surgery, mFRI, SOFA and AKI stage (Table 2, and Fig. S3 showed logistic regression feature importance).

**Table 2:** Results of multivariable analyses

Intercept and Variable	β Coefficient	OR (95% CI)	*P* Value
Baseline eGFR (mL/min/1.73m^2^)			
> 90	NA	NA	
60–89	0.102	1.108 (0.665–1.846)	0.695
30–59	0.506	1.658 (0.965–2.847)	0.037
Female	0.555	1.742 (1.169–2.596)	0.006
AKI stage at enrollment	0.572	1.773 (1.103–2.848)	0.018
Aortic surgery	1.056	2.875 (1.583–5.222)	0.001
SOFA at enrollment	0.201	1.223 (1.131–1.322)	0.000
mFRI (mL/(mg·kg)/2 h)			
> 0.33	NA	NA	
0.22–0.32	0.514	1.672 (0.677–4.153)	0.268
0.14–0.21	0.989	2.690 (1.164–6.218)	0.021
≤ 0.13	2.272	9.706 (4.557–20.652)	0.000
Constant	− 6.691		

The predicted probability of composite outcomes was calculated as:

p = 1/(1 + exp(−(− 6.691 + 0.555*(if female) + 0.102*(if 60 ≤ eGFR < 90) + 0.506*(if 30 ≤ eGFR < 60) + 1.056*(if Aortic surgery) + 2.27*(if mFRI ≤ 0.13) + 0.989*(if 0.14 ≤ mFRI < 0.22) + 0.514*(if 0.22 ≤ mFRI < 0.33) + 0.572*(if AKI stage2 at enrollment) + 0.201*(SOFA at enrollment))))。

Except for SOFA which is a continuous variable, the remaining 5 predictors as binary variables (1 or 0) are included in risk model

GFR, Glomerular Filtration Rate; CABG, Coronary Artery Bypass Grafting; SOFA, Sequential Organ Failure Assessment; mFRI, modified Furosemide Responsiveness Index; CI, confidence interval; NA, not applicable; OR, odds ratio

**Fig. 1:**
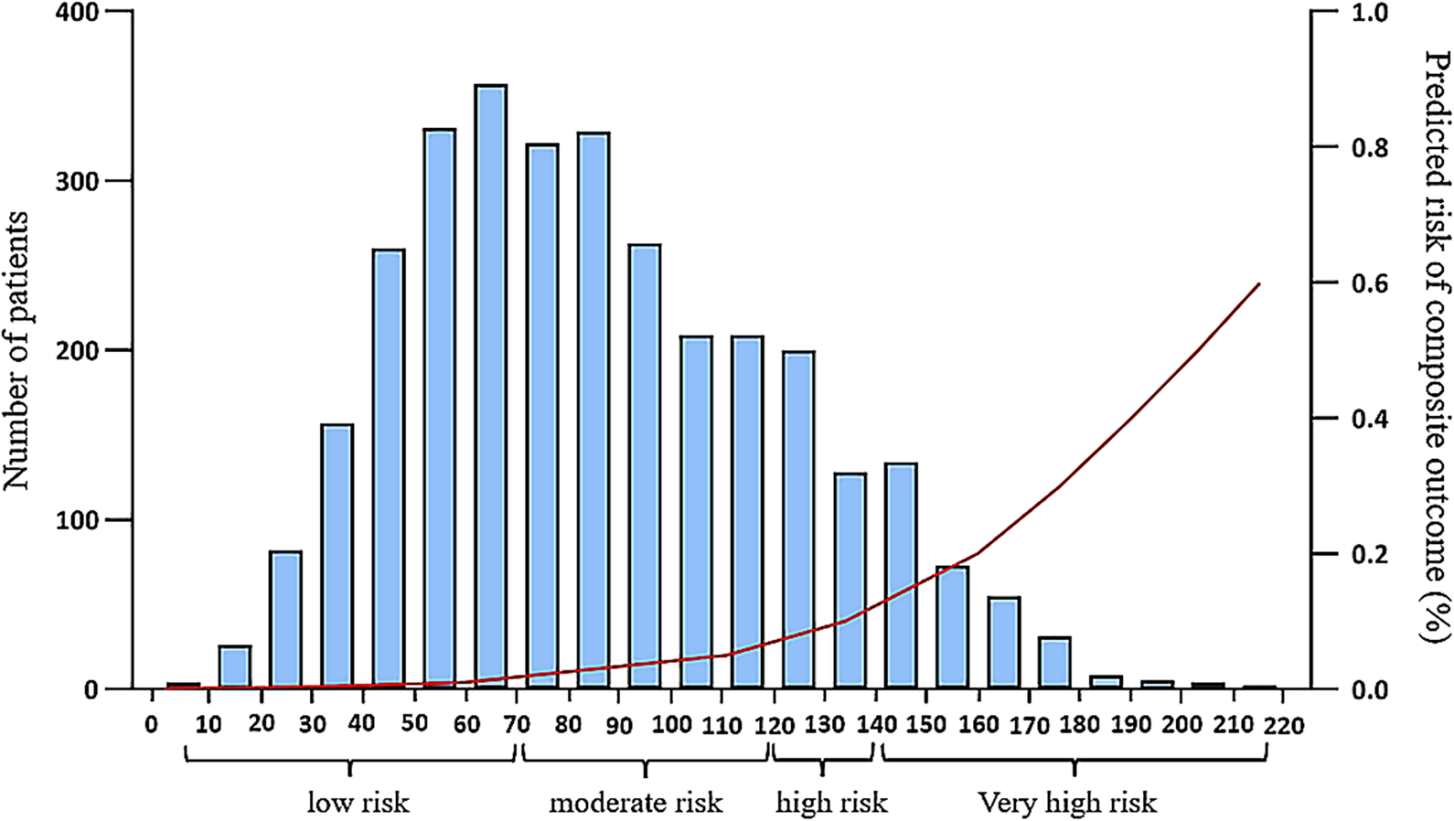
Risk of the composite outcome in the derivation cohort according to risk score values from established Model. Bars show number of patients (left axes) and lines show the predicted risk of composite outcomes (right axes)

The above model was subsequently translated into a risk score: the AKI-Pro score (Table 3), and the score was constructed based on the coefficients from the logistic model. The incidence of composite outcome showed significant increments with escalating risk score assignments. Patients in the derivation cohort were stratified into four groups according to the integer risk score based on the model: low risk (≤ 70 points, n = 1237), moderate risk (71–120 points, n = 1331), high risk (121–140 points, n = 325), and very high risk (> 140 points, n = 295) (Fig. 1). The corresponding observed composite outcome rates were 1.1%, 2.2%, 7.4%, and 17.6%, respectively.

**Table 3:** The AKI-Pro score and risk stratification

Variables	Points	Variables	Points	Variables	Points
Baseline eGFR group		SOFA score at enrollment			
** ≥ **90	0	1	6	2	13
60–89	3	3	19	4	25
30–59	16	5	31	6	38
Female	17	7	44	8	50
AKI stage at enrollment		9	56	10	63
Stage 1	0	11	69	12	75
Stage 2	18	13	81	14	88
Aortic surgery	33	15	94	16	100
mFRI group		17	106	18	113
> 0.33	0	19	119	20	125
0.22–0.32	16	21	131	22	138
0.14–0.21	31	23	144	24	150
≤ 0.13	71				

AKI-Pro Score	Risk group				
> 140	Very high risk				
121–140	High risk				
71–120	Moderate risk				
≤ 70	Low risk				

GFR, Glomerular Filtration Rate; SOFA,  Sequential Organ Failure Assessment; mFRI, modified Furosemide Responsiveness Index

**Fig. 2:**
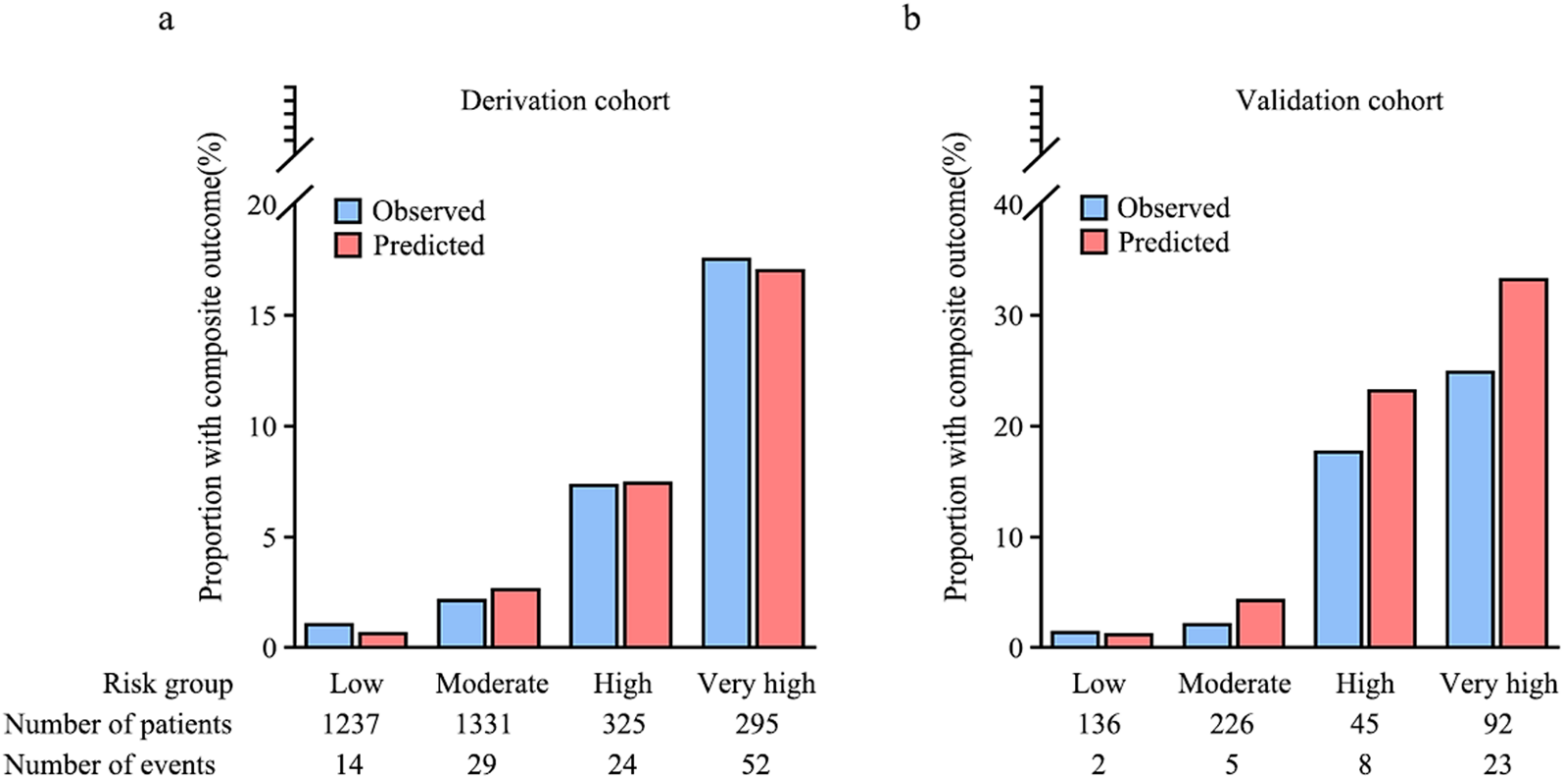
The AKI-Pro score calibration of the derivation (**a**) and validation (**b**) cohort

The AKI-Pro score presented good performance in the discrimination and calibration analysis using the area under the receiving operating characteristic (ROC) curve, with the AUC of 0.79 (95% CI 0.75–0.84) (Fig. S4) and Hosmer–Lemeshow’s goodness-of-fit test (χ^2^ = 5.34, p = 0.17) with non-significant difference between the predicted and observed risk in all ranges for the development of scoring system (Fig. 2a).

The clinical application of risk groups will identify high-risk patients based on a range of predicted risk thresholds for composite outcome (Table S2). For instance, at AKI-Pro score higher than 140 points, 9.3% of patients were categorized as very high risk for progression to severe AKI or death within seven days among early AKI patients after cardiac surgery. The corresponding positive predictive values were 18% and the negative predictive values were 98%.

### External validation

The AKI-Pro score was prospectively validated in the Zhongshan cohort of 499 patients recruited by our institution. The corresponding observed composite outcomes in each risk group were 1.5%, 2.2%, 17.8%, and 25%, respectively. The AKI-Pro score also presented an excellent discrimination in the new data set, with an AUC of 0.84 (95% CI 0.77–0.91) (Fig. S4b). Calibration analysis according to the Hosmer–Lemeshow’s goodness-of-fit test (χ^2^ = 8.36, p = 0.12) also did not demonstrate significant differences in risk groups (Fig. 2b).

**Fig. 3:**
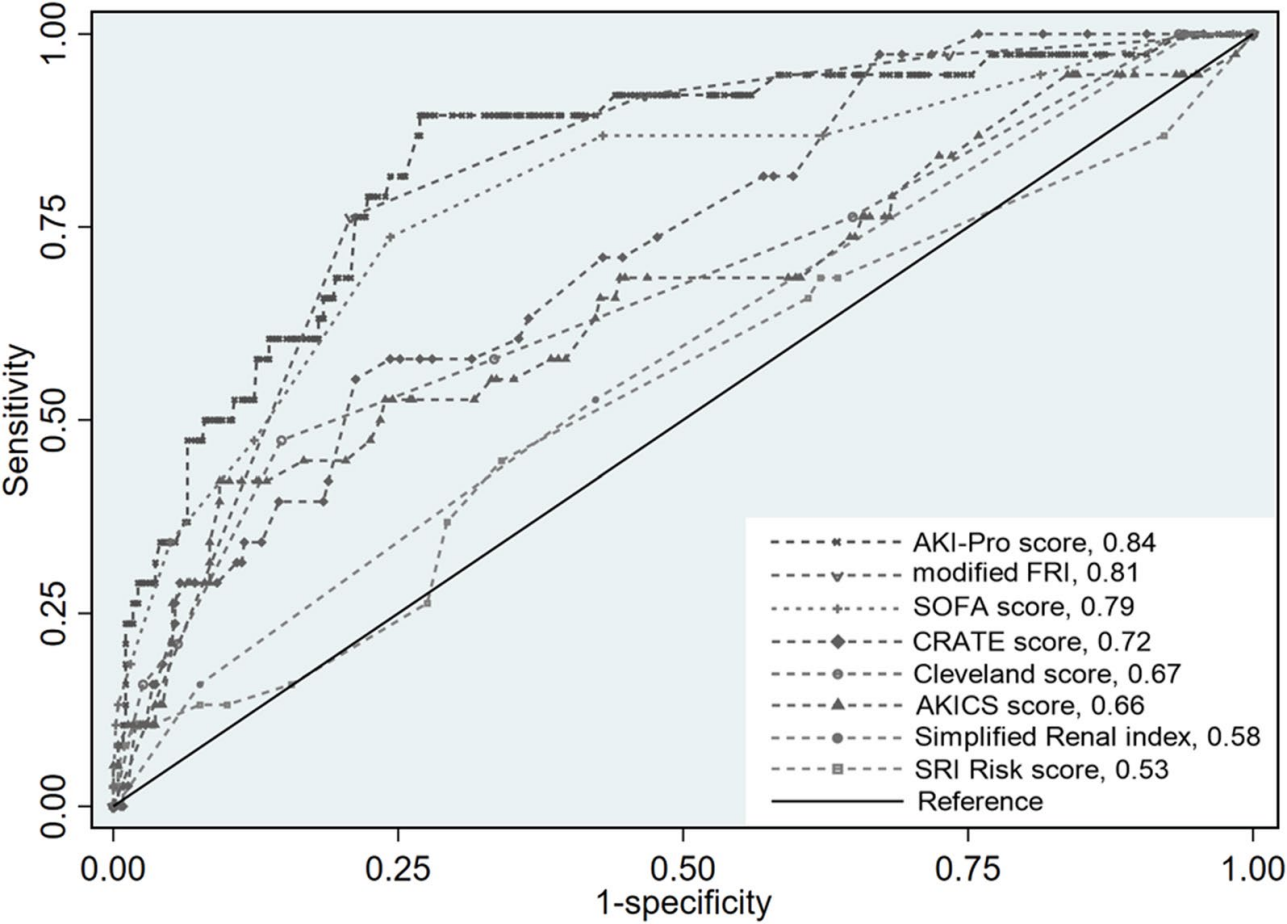
Model comparison between the AKI-Pro score and other established risk scores

We also compared the discrimination of AKI-Pro score to the SOFA [14], CRATE score [15], Cleveland score [16], AKICS score [17], Simplified renal index [18], and SRI risk score [19] for the composite outcome in the validation cohort. The AUROC for the AKI-Pro score (0.84 [95% CI 0.77–0.91]) was better than those of the CRATE score(0.72 [95% CI 0.64–0.80], *P* = 0.0024), Cleveland score(0.67 [95% CI 0.57–0.76], *P* = 0.0003), AKICS score(0.66 [95% CI 0.55–0.76], *P* = 0.0013), Simplified renal index (0.58[95% CI 0.49–0.66], *P* = 0.000), and SRI risk score(0.53[95% CI 0.43–0.63], *P* = 0.000) (Fig. 3 and Table S3). Reliable AUC calculation of established risk score was not realistic by using the derivation cohort due to partial data unavailable in the MIMIC-IV database.

### Clinical utility

Decision curve analysis (DCA) was used to evaluate the clinical utility value of our risk model. The DCA curves showed that if the threshold probability is 5–50%, using the risk model in the current study to predict the composite outcome risk could add more benefit (Fig. S5). However, whether a model leads to changes in clinical decision would control the occurrence of outcomes and clinical benefits needs further verification.

## Discussion

In the present study, we developed and externally validated a new risk score, the AKI-Pro score, to predict patients who experienced mild/moderate AKI progression to the composite outcome. The AKI-Pro score, including six readily available clinical variables, demonstrated good performance with discrimination and calibration for the composite outcome. We also found that the predictive ability of AKI-Pro score was prior to previously established scoring system in predicting AKI progression to severe AKI, including dialysis or death within seven days following cardiac surgery. Notably, the scoring system is easy to calculate and can be applied to most medical institutions.

Furosemide, a commonly used medication in cardiac surgery patients with fluid overload, exerts its pharmacological effects by actively secreting into the luminal space of proximal tubules and inhibiting the sodium-chloride-potassium (Na^+^/K^+^/2Cl^−^) co-transporter at the ascending limb of the loop of Henle. This mechanism leads to the inhibition of sodium reabsorption, resulting in increased natriuresis and urine output [20]. Due to impaired renal tubular function, patients with AKI often exhibit a diminished diuretic response to furosemide. Consequently, the diuretic response has been recognized as a cost-effective and straightforward method for assessing tubular damage during AKI. The FST has been proposed as a reliable indicator for predicting the progression of AKI to severe AKI or the need for RRT in the early stages of AKI [21, 22]. Nonetheless, concerns have been raised regarding potential adverse effects associated with FST, such as hypovolemia, which restricts its broad application in critically ill patients. To address this limitation, a simplified version of FST, known as the furosemide responsiveness index (FRI), has been proposed as a surrogate measure [23]. In our previous study, we further refined the FRI and conducted two distinct exploratory analyses to validate the predictive value of the modified FRI (mFRI) for AKI progression to severe AKI. Consequently, the model analysis incorporated mFRI as a pivotal variable.

The SOFA score has gained widespread recognition as a valuable tool for assessing organ dysfunction in critically ill patients. Initially designed to quantify the severity of organ dysfunction and predict outcomes in septic patients, the SOFA score has found applicability across a range of critical conditions [24]. Our findings revealed a statistically significant association between higher SOFA scores and an increased risk of unfavorable renal outcomes. AKI is commonly observed as a concurrent or sequential manifestation in the presence of sepsis, shock, trauma, or other pathological conditions [25]. The progression to severe AKI or death is influenced not only by the extent of renal insult but also by the degree of overall organ dysfunction [26]

Aortic surgery also serves as a significant contributor to adverse renal composite outcome in the AKI-Pro Score. Type A Aortic Dissection (ATAAD) represents a prevalent form of aortic surgery and is particularly susceptible to complications, including hypovolemic shock and organ hypoperfusion affecting vital organs such as the heart, brain, kidney, and intestine [27]. A recent study has demonstrated that AKI is a frequent postoperative complication following ATAAD surgery and independently predicts unfavorable long-term outcomes [28].

Previous studies reported that female is an independent risk factor for perioperative AKI [16, 19]. We have observed significant interactions between female gender and FR parameters in predicting AKI progression compared to males [11]. In the present model, the female gender combined with other variables contributed to the prediction ability for composite outcomes. Our analysis also revealed that baseline eGFR and the stage of AKI at the time of enrollment emerged as significant variables influencing the progression of AKI to stage 3 or mortality. These findings align with previous studies that have consistently demonstrated pre-existing chronic kidney disease, as indicated by reduced eGFR, to be a robust risk factor for AKI, dialysis, and mortality [29–31]. Furthermore, the AKI stage at enrollment appeared to reflect the severity of renal insult and exhibited a definite association with unfavorable outcomes.

Several risk stratification scoring systems, including the CRATE score, Cleveland score, AKICS score, Simplified Renal Index, and SRI risk score, have been previously developed to forecast the likelihood of AKI or the need for dialysis after cardiac surgery [15–19]. In this study, we also compare the predictive performance of these aforementioned scoring systems with the AKI-Pro score in the validation cohort, specifically concerning poor renal outcomes. Our findings demonstrated that the AKI-Pro score exhibited superior predictive capability compared to the classical risk scores in forecasting the composite outcome during early stages of AKI following cardiac surgery.

This study also has several limitations: First, although the model showed favorable discrimination, there is still low-density distribution of patients with composite outcome in the population, and the predictive risk in high-risk patients may be overestimated, resulting in bias. Second, given that this study only included patients following cardiac surgery, the predictive value of AKI-Pro score in other AKI entities, such as contrast-induced AKI and sepsis-associated AKI should be explored. Third, we could not include intraoperative risk factors such as CPB time, cross-clamp time, hemodilution, intraoperative hypotension and fluid management because the MIMIC-IV database on these variables was not available. Last, we mainly focused on early diagnosed mild/moderate AKI progression after surgery. Patients with no AKI or severe AKI within 48 h after the procedure were not enrolled in our population.

## Conclusions

We introduced a novel predictive score (AKI-Pro score) to identify the high risk of progression to severe AKI or death in patients with mild/moderate AKI after cardiac surgery. The proposed risk score holds promise as a valuable tool for clinicians, enabling early identification of high-risk individuals and facilitating optimal clinical decision-making during hospitalization.

### Supplementary Information

Additional file 1: Table S1. Bivariate analyses of study variables versus composite outcome for derivation and validation cohort. Table S2. Proportion of patients at risk groups who are considered for intervention, and corresponding sensitivity, specificity, positive and negative predictive values. Table S3. Model comparison between the AKI-Pro score and other established risk scores.

Additional file 2: Fig S1. Flow chart of the study.

Additional file 3: Fig S2. Predictor selection using the LASSO regression method.

Additional file 4: Fig S3. Logistic regression feature importance.

Additional file 5: Fig S4. Model performance of ROC curve in the derivation and validation cohort.

Additional file 6: Fig S5. Decision curve analysis of the clinical utility value of the risk model.

## Data Availability

If the request is reasonable, the corresponding author can share the data from this study to the requestor.

## Abbreviations

AKI: Acute kidney injury
RRT: Renal replacement therapy
MIMIC-IV: Medical Information Mart for Intensive Care IV
Scr: Serum creatinine
GFR: Glomerular filtration rate
FST: Furosemide stress test
mFRI: Modified furosemide responsiveness index
SOFA: Sequential Organ Failure Assessment
BMI: Body mass index
DM: Diabetes mellitus
IHD: Ischemic heart disease
COPD: Chronic obstructive pulmonary disease
CABG: Coronary artery bypass grafting
CKD: Chronic kidney disease
ESRD: End‑stage renal disease
ATAAD: Type A aortic dissection
ICU: Intensive care unit
KDIGO: Kidney disease improving global outcomes
MDRD: Modification of diet in renal disease
IL‑18: Interleukin‑18
NGAL: Neutrophil gelatinase‑associated lipocalin
L-FABP: L-type fatty acid binding protein
CysC: Cystatin C
TIMP‑2: Tissue inhibitor of metalloproteinase‑2
IGFBP7: Insulin like growth factor‑binding protein‑7
LASSO: Least Absolute Shrinkage and Selection Operator
AUC: Area Under the Curve
ROC: Receiver operating characteristic
DCA: Decision curve analysis
NB: Net benefit
CI: Confidence interval
OR: Odds ratio
SD: Standard deviation
