# Supplementary material for: AKI-Pro score for predicting progression to severe acute kidney injury or death in patients with early acute kidney injury after cardiac surgery
Source: J Transl Med. 2024 Jun 16;22:571. doi: 10.1186/s12967-024-05279-4 (PMC11180399; doi:10.1186/s12967-024-05279-4)
Supplement: Supplementary file 1 — Additional file 1: Table S1. Bivariate analyses of study variables versus composite outcome for derivation and validation cohort. Table S2. Proportion of patients at risk groups who are considered for intervention, and corresponding sensitivity, specificity, positive and negative predictive values. Table S3. Model comparison between the AKI-Pro score and other established risk scores. [file 12967_2024_5279_MOESM1_ESM.docx]

# Supplementary material

**Fig S1. Flow chart of the study** (MIMIC = Medical Information Mart for Intensive Care; AKI = Acute Kidney Injury; CKD = Chronic Kidney Disease)

**Fig S2. Predictor selection using the LASSO regression method. (a)** A coefficient profile plot was produced against the log (λ) sequence. The dotted vertical lines were plotted at the optimal values using the minimum criteria and the one standard error of the minimum criteria (the 1−SE criteria). Predictors were selected based on the 1−SE criteria. **(b)** Ten-fold cross-validation via minimum criteria was used to select the Tuning parameter (λ) in the LASSO model.

**Fig S3. Logistic regression feature importance.**

**Fig S4. Model performance of ROC curve in the derivation and validation cohort.**

**Fig S5. Decision curve analysis of the clinical utility value of the risk model in the derivation and validation cohort.**

| Table S1. Bivariate analyses of study variables versus composite outcome for derivation and validation cohort | | | | | | |
| --- | --- | --- | --- | --- | --- | --- |
|  | Derivation cohort | |  | Validation cohort | |  |
|  | Non-composite outcome (n=3069) | Composite outcome (n=119) | *P* value | Non-composite outcome (n=461) | Composite outcome (n=38) | *P* value |
| Age, years mean (IQR) | 70 (62-77) | 72 (66-78) | 0.03 | 61 (62-77) | 59 (66-78) | 0.31 |
| Gender, (%) |  |  | 0.02 |  |  | 0.22 |
| Famale | 925 (30.1) | 48 (40.3) |  | 107 (23.2) | 5 (13.2) |  |
| Male | 2144 (69.9) | 69 (59.7) |  | 354 (76.8) | 33 (86.8) |  |
| BMI_category, kg/m^2^, n(%) | |  | 0.53 |  |  | 0.01 |
| 25.0-29.9 | 972 (31.7) | 36 (30.3) |  | 263 (57.0) | 19 (50.0) |  |
| <24.9 | 380 (12.4) | 11 (9.2) |  | 168 (36.4) | 11 (28.9) |  |
| ≥30 | 1717 (55.9) | 72 (60.5) |  | 30 (6.5) | 8 (21.1) |  |
| Comorbidities |  |  |  |  |  |  |
| Hypertension, n(%) |  |  | 0.24 |  |  | 0.40 |
| No | 607 (19.8) | 18 (15.1) |  | 205 (44.5) | 20 (52.6) |  |
| Yes | 2462 (80.2) | 101 (84.9) |  | 256 (55.5) | 18 (47.4) |  |
| Diabetes mellitus, n(%) |  |  | 0.37 |  |  | 0.34 |
| No | 2376 (77.4) | 88 (73.9) |  | 390 (84.6) | 35 (92.1) |  |
| Yes | 693 (22.6) | 31 (26.1) |  | 71 (15.4) | 3 (7.9) |  |
| Ischemic heart disease, n(%) | |  | 0.66 |  |  | 1.00 |
| No | 697 (22.7) | 29 (24.4) |  | 406 (88.1) | 34 (89.5) |  |
| Yes | 2372 (77.3) | 90 (75.6) |  | 55 (11.9) | 4 (10.5) |  |
| COPD, n(%) |  |  | 0.87 |  |  | 1.00 |
| No | 2776 (90.5) | 109 (91.6) |  | 459 (99.6) | 38 (100.0) |  |
| Yes | 293 (9.5) | 10 (8.4) |  | 2 (0.4) | 0 (0.0) |  |
| Baseline eGFR group, n(%) | |  | <0.01 |  |  | 0.05 |
| >90 | 1016 (33.1) | 24 (20.2) |  | 127 (27.5) | 4 (10.5) |  |
| 60-90 | 1468 (47.8) | 50 (42.0) |  | 244 (52.9) | 26 (68.4) |  |
| 30-60 | 585 (19.1) | 45 (37.8) |  | 90 (19.5) | 8 (21.1) |  |
| Exposure to at least one nephrotoxic agent, n (%) |  |  | 0.09 |  |  | 0.21 |
| No | 2378 (77.5) | 84 (70.6) |  | 305 (66.2) | 21 (55.3) |  |
| Yes | 691 (22.5) | 35 (28.4) |  | 156 (33.8) | 17 (44.7) |  |
| Preoperative diuretic exposure, n (%) |  |  | 0.47 |  |  | 0.30 |
| No | 2507 (81.7) | 101 (84.9) |  | 116 (36.0) | 17 (44.7) |  |
| Yes | 562 (18.3) | 18 (15.1) |  | 156 (64.0) | 21 (55.3) |  |
| Type of surgery, n(%) |  |  | <0.01 |  |  | 0.07 |
| CABG only | 1586(51.7) | 50 (42.0) |  | 52 (11.3) | 4 (10.5) |  |
| Valve only | 728 (23.7) | 24 (20.2) |  | 273 (59.2) | 15 (39.5) |  |
| CABG and valve | 527 (17.2) | 25 (21.8) |  | 30 (6.5) | 4 (10.5) |  |
| Aortic surgery | 167 (5.4) | 16 (13.4) |  | 82 (17.8) | 11 (28.9) |  |
| Others | 61 (2.0) | 3 (2.5) |  | 24 (5.2) | 4 (10.5) |  |
| AKI stage, (%) |  |  | <0.01 |  |  | <0.01 |
| Stage 1 | 1267 (41.3) | 24 (20.2) |  | 421 (91.3) | 21 (55.3) |  |
| Stage 2 | 1802 (58.7) | 95 (79.8) |  | 40 (8.7) | 17 (44.7) |  |
| Hemoglobin below 9g/dL, n (%) |  |  | 0.02 |  |  | 1.00 |
| No | 2094 (68.2) | 69 (58.0) |  | 342 (74.2) | 28 (73.7) |  |
| Yes | 975 (31.8) | 50 (42.0) |  | 119 (25.8) | 10 (26.3) |  |
| SOFA score, mean (IQR) | 5.0 (4.0, 7.0) | 7.0 (5, 9.0) | <0.01 | 8.0 (7, 9) | 10 (9, 12) | <0.01 |
| Vasopressor use, n (%) |  |  | <0.01 |  |  | <0.01 |
| No | 2677 (87.2) | 71 (59.7) |  | 202 (43.8) | 6 (15.8) |  |
| Yes | 392 (12.8) | 48 (40.3) |  | 259 (56.2) | 32 (84.2) |  |
| mFRI, mean (IQR) | 0.2 (0.1, 0.3) | 0.1 (0, 0.2) | <0.01 | 0.2 (0.1, 0.3) | 0.1 (0, 0.1) | <0.01 |
| mFRI group, (%) |  |  | <0.01 |  |  | <0.01 |
| ≤0.13 | 713 (23.2) | 79 (66.4) |  | 96 (20.8) | 29 (76.3) |  |
| 0.14-0.21 | 780 (25.4) | 20 (16.8) |  | 119 (25.8) | 6 (15.8) |  |
| 0.22-0.32 | 786 (25.6) | 12 (10.1) |  | 123 (26.7) | 2 (5.3) |  |
| ≥0.33 | 790 (25.7) | 8 (6.7) |  | 123 (26.7) | 1 (2.6) |  |
| *COPD = Chronic Obstructive Pulmonary Disease; GFR= Glomerular Filtration Rate; CABG = Coronary Artery Bypass Grafting; SOFA = Sequential Organ Failure Assessment; mFRI = modified Furosemide Responsiveness Index.*  Categorical variables were presented as frequency rates and percentages; continuous variables were expressed median (IQR). | | | | | | |
|  | | | | | |  |

**Table S2.** Proportion of patients at risk groups who are considered for intervention, and

corresponding sensitivity, specificity, positive and negative predictive values.

|  | Predicted risk category | | |
| --- | --- | --- | --- |
|  | >70 | >120 | >140 |
| No. of patients (%) | 1951(62) | 620(20) | 295(9.3) |
| Sensitivity | 0.88 | 0.64 | 0.44 |
| Specificity | 0.40 | 0.82 | 0.92 |
| Positive Predictive Value | 0.05 | 0.12 | 0.18 |
| Negative Predictive Value | 0.99 | 0.98 | 0.98 |

Table S3. Model comparisons between the AKI-Pro score and other established risk scores.

|  | Composite outcome  AUC | SE | 95% CI |
| --- | --- | --- | --- |
| AKI-Pro score | 0.8411 | 0.0360 | 0.7705-0.9117 |
| mFRI | 0.8129 | 0.0304 | 0.7534-0.8724 |
| SOFA score [14] | 0.7874 | 0.0417 | 0.7057-0.8691 |
| The CRATE score  (Tamayo 2016) [15] | 0.7173 | 0.0401 | 0.6388-0.7959 |
| Cleveland Clinic score  (Thakar 2005) [16] | 0.6656 | 0.0504 | 0.5668-0.7644 |
| AKICS score  (Palomba 2007) [17] | 0.6552 | 0.0526 | 0.5521-0.7582 |
| Simplified Renal index  (Wijeysundera 2007) [18] | 0.5777 | 0.0441 | 0.4913-0.6642 |
| SRI (Severe Renal Insufficiency) Risk score (Brown 2007) [19] | 0.5307 | 0.0515 | 0.4299-0.6316 |
| chi2(3) =61.56 Prob>chi2 =0.0000  *AUC= Area Under the Curve; SE= Standard Erro; CI= Confidence Interval.* | | | |
