# Supplementary figures and images for: AKI-Pro score for predicting progression to severe acute kidney injury or death in patients with early acute kidney injury after cardiac surgery
Source: J Transl Med. 2024 Jun 16;22:571. doi: 10.1186/s12967-024-05279-4 (PMC11180399; doi:10.1186/s12967-024-05279-4)

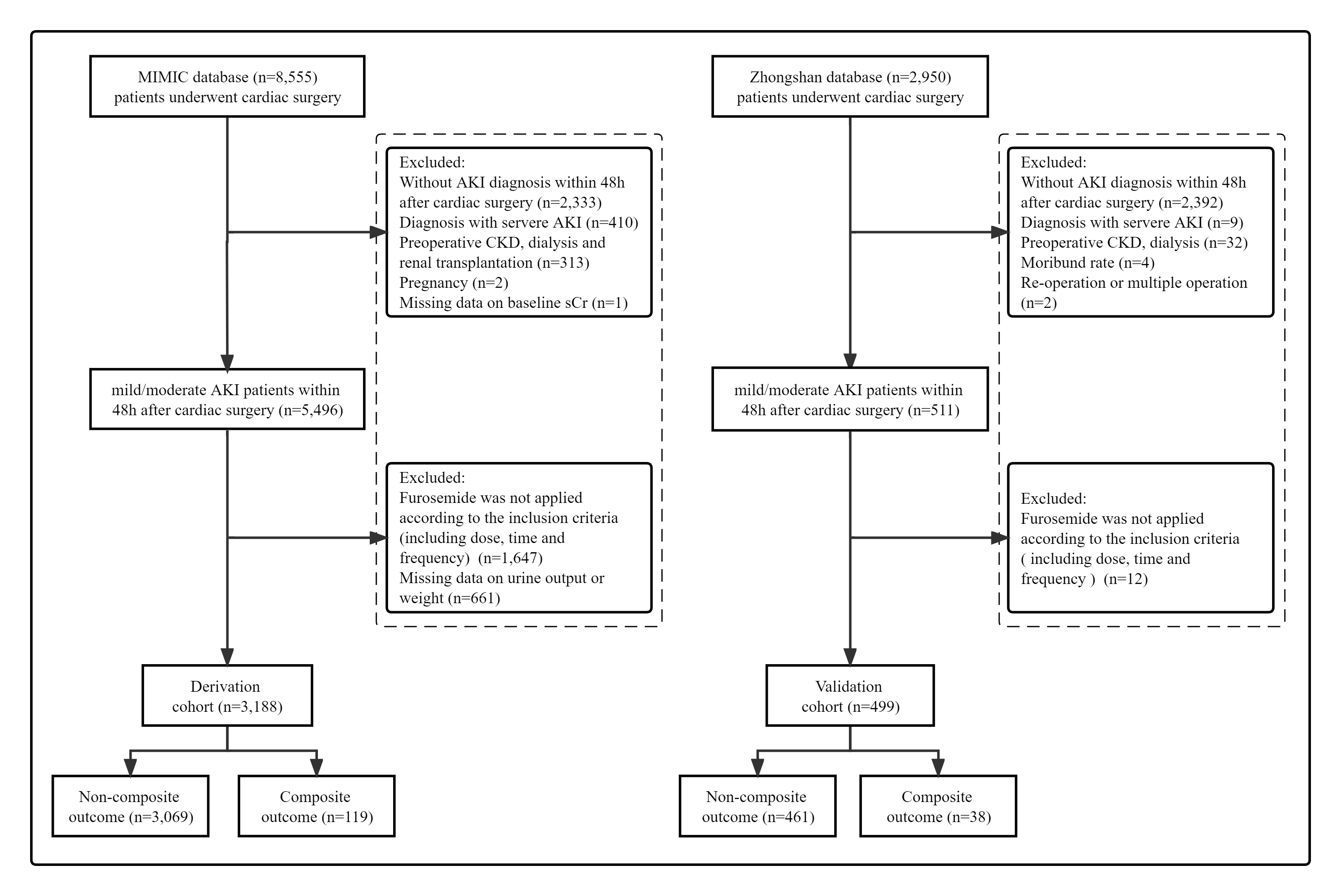

Supplement: Supplementary file 2 — Additional file 2: Fig S1. Flow chart of the study. [file 12967_2024_5279_MOESM2_ESM.png]

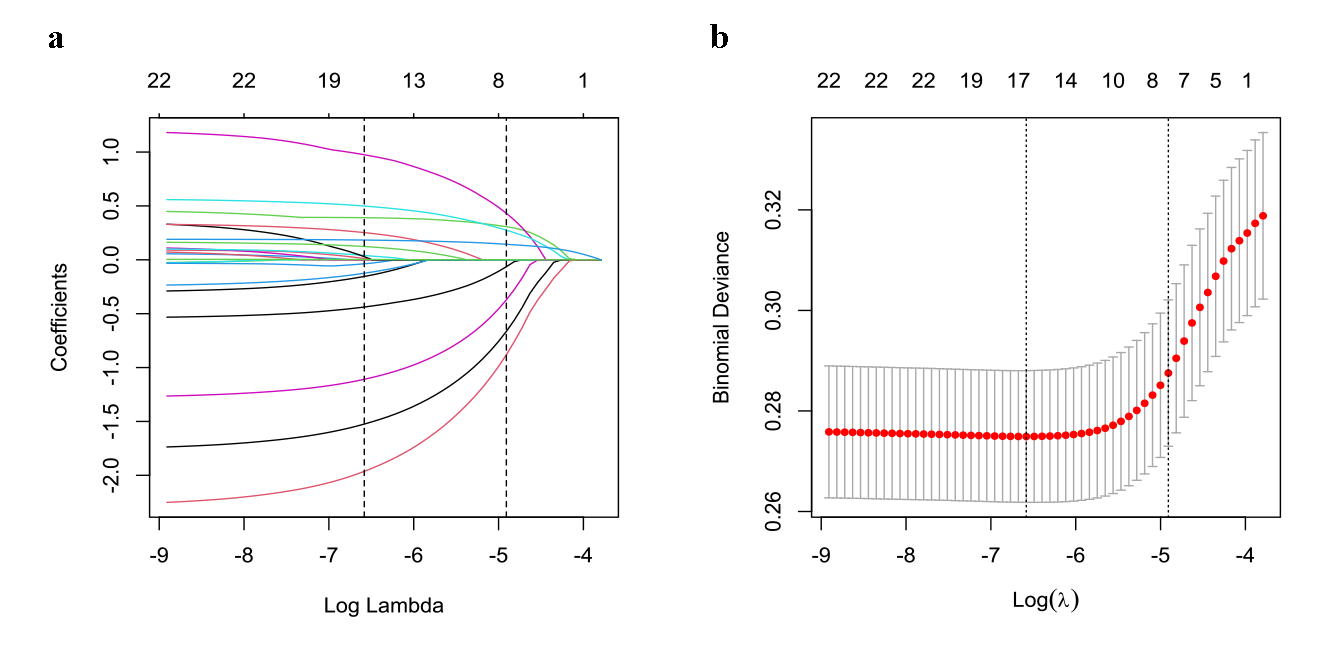

Supplement: Supplementary file 3 — Additional file 3: Fig S2. Predictor selection using the LASSO regression method. [file 12967_2024_5279_MOESM3_ESM.png]

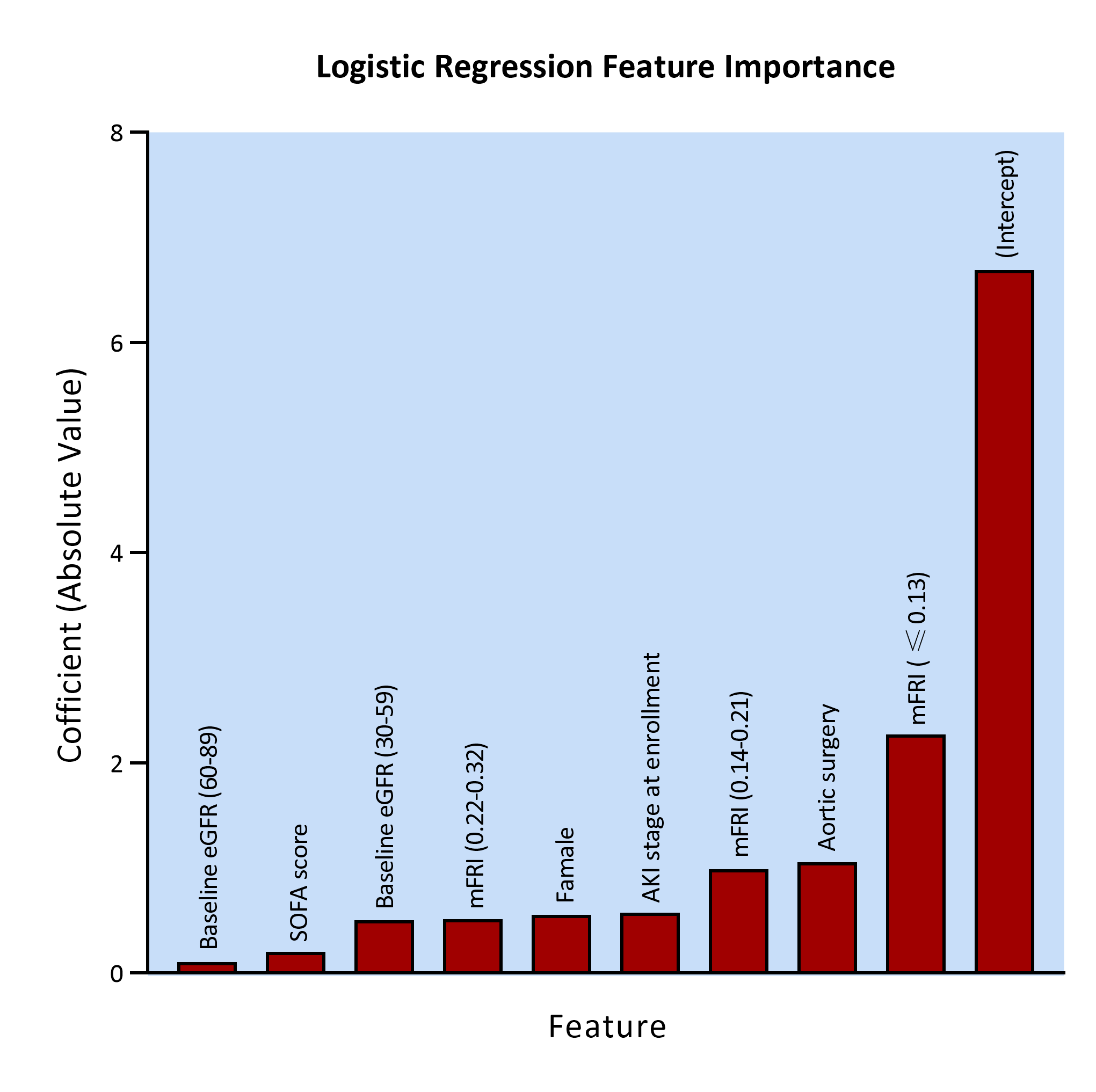

Supplement: Supplementary file 4 — Additional file 4: Fig S3. Logistic regression feature importance. [file 12967_2024_5279_MOESM4_ESM.png]

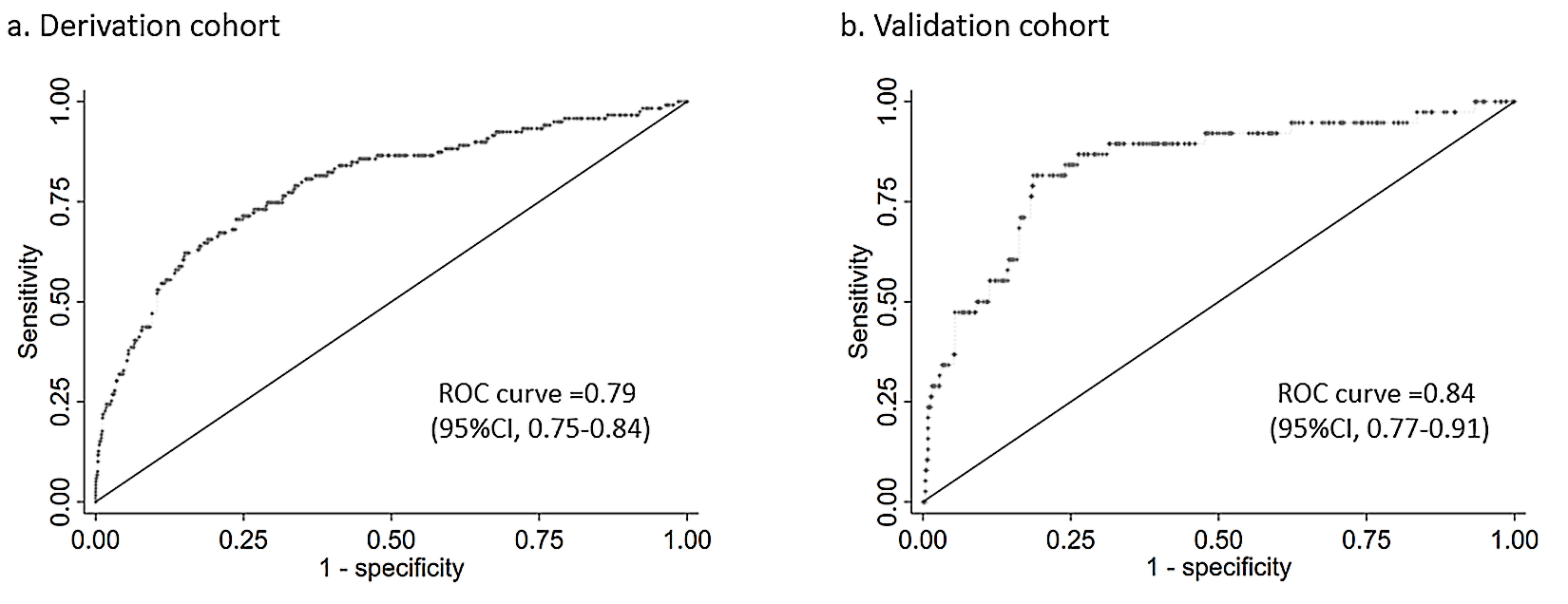

Supplement: Supplementary file 5 — Additional file 5: Fig S4. Model performance of ROC curve in the derivation and validation cohort. [file 12967_2024_5279_MOESM5_ESM.png]

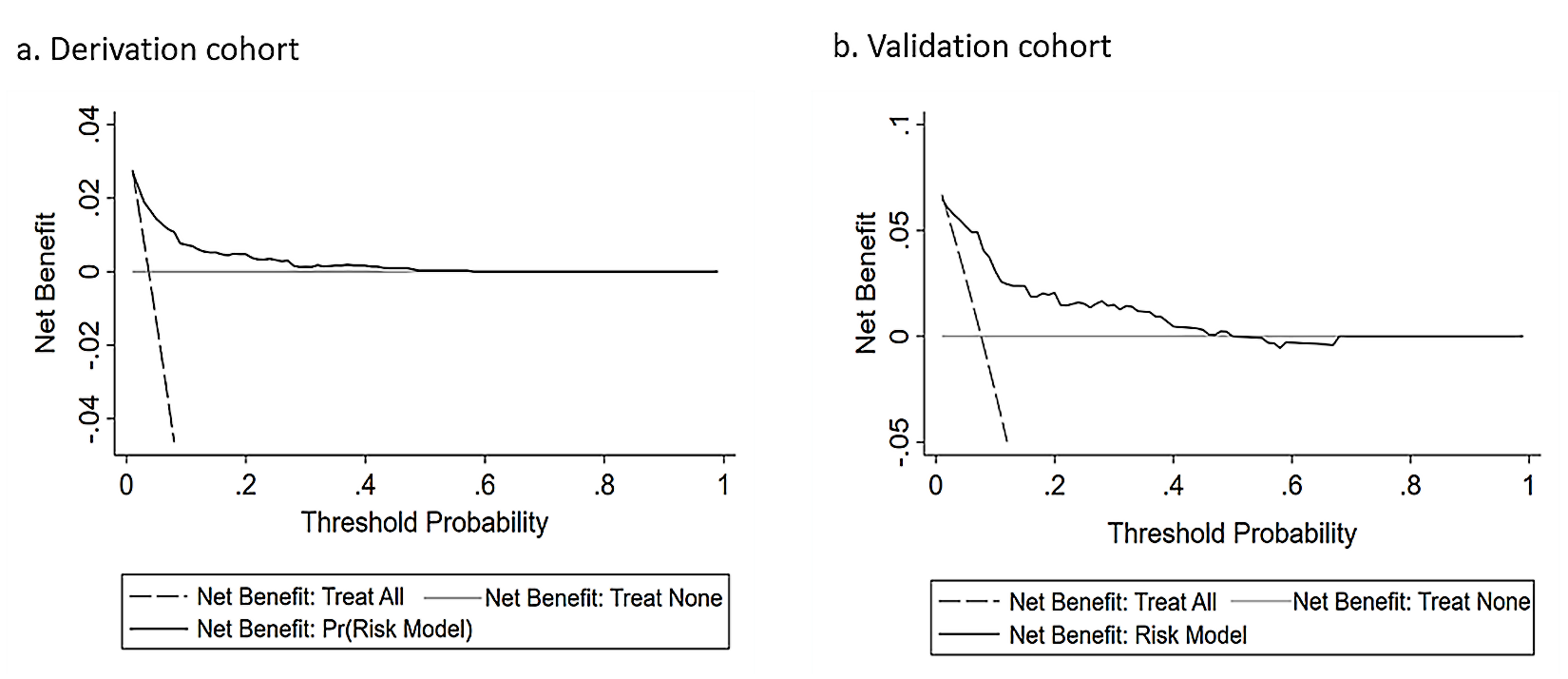

Supplement: Supplementary file 6 — Additional file 6: Fig S5. Decision curve analysis of the clinical utility value of the risk model. [file 12967_2024_5279_MOESM6_ESM.png]
